# Endolysosomal Cation Channels and Lung Disease

**DOI:** 10.3390/cells11020304

**Published:** 2022-01-17

**Authors:** Barbara Spix, Aicha Jeridi, Meshal Ansari, Ali Önder Yildirim, Herbert B. Schiller, Christian Grimm

**Affiliations:** 1 Walther Straub Institute of Pharmacology and Toxicology, Faculty of Medicine, Ludwig-Maximilians-University, 80336 Munich, Germany; barbara.spix@cup.uni-muenchen.de; 2 Pneumology Center, Institute of Lung Biology and Disease, Helmholtz Zentrum München, 85764 Munich, Germany; aicha.jeridi@helmholtz-muenchen.de (A.J.); meshal.ansari@helmholtz-muenchen.de (M.A.); oender.yildirim@helmholtz-muenchen.de (A.Ö.Y.); herbert.schiller@helmholtz-muenchen.de (H.B.S.)

**Keywords:** TRPML, TRPML3, TRPA1, TRPM2, TRPV2, BK, emphysema, lung injury, COPD, asthma, cystic fibrosis

## Abstract

Endolysosomal cation channels are emerging as key players of endolysosomal function such as endolysosomal trafficking, fusion/fission, lysosomal pH regulation, autophagy, lysosomal exocytosis, and endocytosis. Diseases comprise lysosomal storage disorders (LSDs) and neurodegenerative diseases, metabolic diseases, pigmentation defects, cancer, immune disorders, autophagy related diseases, infectious diseases and many more. Involvement in lung diseases has not been a focus of attention so far but recent developments in the field suggest critical functions in lung physiology and pathophysiology. Thus, loss of TRPML3 was discovered to exacerbate emphysema formation and cigarette smoke induced COPD due to dysregulated matrix metalloproteinase 12 (MMP-12) levels in the extracellular matrix of the lung, a known risk factor for emphysema/COPD. While direct lung function measurements with the exception of TRPML3 are missing for other endolysosomal cation channels or channels expressed in lysosome related organelles (LRO) in the lung, links between those channels and important roles in lung physiology have been established such as the role of P2X4 in surfactant release from alveolar epithelial Type II cells. Other channels with demonstrated functions and disease relevance in the lung such as TRPM2, TRPV2, or TRPA1 may mediate their effects due to plasma membrane expression but evidence accumulates that these channels might also be expressed in endolysosomes, suggesting additional and/or dual roles of these channels in cell and intracellular membranes. We will discuss here the current knowledge on cation channels residing in endolysosomes or LROs with respect to their emerging roles in lung disease.

## 1. Introduction

The five major human lung diseases comprise chronic obstructive pulmonary disease (COPD), asthma, acute lower respiratory tract infections, tuberculosis and lung cancer. COPD is the most prevalent chronic lung disease. It is a global health issue, affecting nearly 300 million people worldwide and resulting in the death of about 3 million individuals each year. COPD is currently the third leading cause of death worldwide. Exposure to tobacco smoke constitutes the leading cause of COPD. Other risk factors include inhalation of occupational dusts and fumes from chemicals, or environmental pollutants such as exhaust gases from vehicles and industries. Due to the still high prevalence of smoking, aging populations and rising air pollution through exhaust gases, the COPD prevalence is likely to increase further in the next few years. Occupational and environmental pollutants as well as certain medications may also cause another toxic chronic lung disease, pulmonary fibrosis. Pulmonary fibrosis is characterized by an irreversible remodeling of normal lung tissue to scarred tissue around and between the air sacs, resulting in an impaired oxygen exchange in the lungs. In contrast to asthma or tuberculosis, there are currently no drugs available that can efficiently treat and/or reduce either COPD or pulmonary fibrosis mortality and there is hence an urgent need for novel treatment approaches and targets.

We will discuss here the recent discovery that a non-selective Ca^2+^ permeable cation channel of the TRP (transient receptor potential) superfamily, TRPML3, which is mainly expressed in alveolar macrophages in the lung and which is residing in intracellular compartments, so called endosomes and lysosomes, results in exacerbation of lung tissue injury and emphysema formation under basal, elastase or cigarette smoke exposure when absent or dysfunctional [1]. In this context, we will also discuss current knowledge on functional roles of other endolysosomal/vesicular/LRO ion channels in the lung.

Sodium, potassium and chloride ion channels and transporters including several TRP channels play critical roles in maintaining lung homeostasis and are associated with a number of human lung diseases including cystic fibrosis (CF), COPD, pulmonary edema, and chronic bronchitis [2]. Cystic fibrosis transmembrane conductance regulator (CFTR), an ATP-gated anion channel, is probably the best known and most intensively studied, disease relevant ion channel in the lung. Mutations in CFTR cause CF, also called mucoviscidosis. CF is the most frequent autosomal recessive disease among Caucasians with an incidence in Europe of about 1:3500 [3]. The most common mutation is F508del which accounts for approximately 66% of the CF chromosomes in the Caucasian population [3]. Recently introduced therapies (CFTR modulators) such as ivacaftor-Kalydeco^®^ or lumacaftor/ivacaftor-Orkambi^®^ [4] are associated with a lower rate of pulmonary exacerbations, hospitalizations and use of antibiotics [4]. Other ion channels such as the Ca^2+^ -activated chloride channel TMEM16A which modulates mucin secretion and airway smooth muscle contraction, and is upregulated in asthmatic patients [2,5] or several TRP channels such as TRPC6, TRPV1, TRPV4, or TRPM6 have been demonstrated to be involved in pathways associated with asthma, CF, emphysema/COPD, lung fibrosis, or edema formation [6]. Thus, e.g., TRPV4 channels are essential for alveolar epithelial barrier function as protection from lung edema [7] and lungs from mice lacking TRPC6 are protected from lung ischaemia-reperfusion-induced oedema (LIRE) [8], while magnesium deficiency in TRPM6 results in severe emphysema [9]. Endolysosomal TRPML channels or mucolipins, which comprise three members in the mammalian genome, TRPML1-3 had not been identified until recently as lung disease relevant. Other confirmed or bona fide endolysosomal/vesicular/LRO cation channels proposed to be associated with lung function that we will discuss here are P2X4, TRPV2, TRPM2, TRPA1, and BK.

## 2. P2X4—Regulator of Surfactant Release: Lamellar Bodies of ATII Cells

P2X purinoceptor 4 (P2X4) belongs to the ligand gated ion channel superfamily, are primarily Na^+^, K^+^, and Ca^2+^ permeable and is activated by adenosine 5′-triphosphate (ATP). P2X4 deficiency in mice reportedly alleviates allergen-induced airway inflammation (AAI) and targeting P2X4 was proposed as a new therapeutic option for allergic asthma [10]. In this work by Zech and colleagues it was further proposed that P2X4 signaling contributes to AAI pathogenesis by regulating dendritic cell mediated Th2 cell priming via modulating IL-1β secretion.

**Figure 1:**
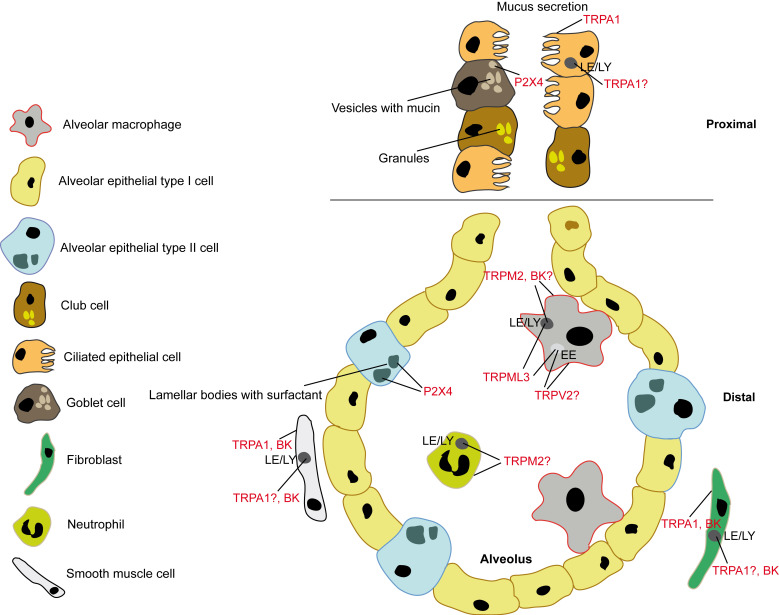
Schematic of an alveolus showing expression of confirmed and putative endolysosomal/vesicular/LRO cation channels involved in lung physiology and disease in the different alveolar/lung cell types. Black labeled compartments represent nuclei.

**Figure 2:**
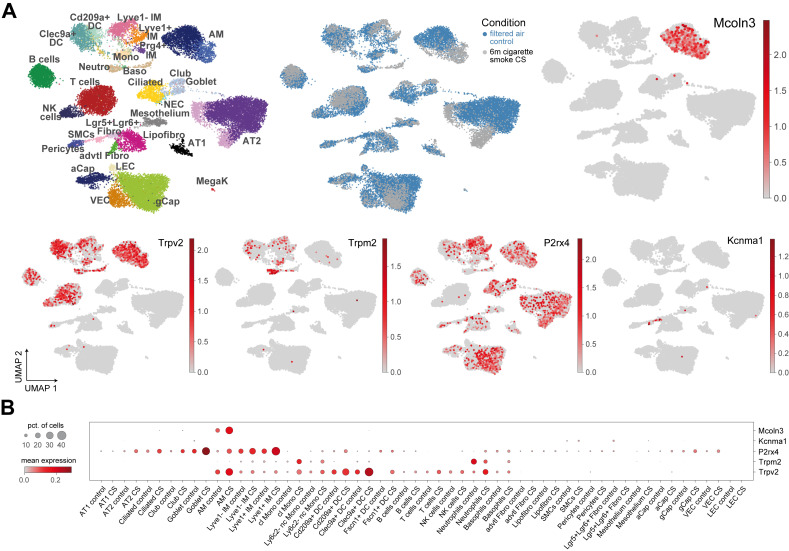
(**A**) Single-cell suspension from murine whole lungs were analyzed using Drop-seq following 6 months of either filtered air (control) or cigarette smoke exposure (CS). The transcriptomes data are projected using the UMAP algorithm, each cell colour-coded by cell type, exposure condition and expression values of indicated genes. Cell types with prominent expression are highlighted. (**B**) The dotplot reflects normalized expression levels of selected genes across cell types. The dot colour indicates the expression level, and the dot size the percentage of cells expressing the gene per group. DC = dendritic cells; IM = interstitial macrophages; Mono = monocytes; AM = alveolar macrophages; Neutro = neutrophils; Baso = basophils; Ciliated = ciliated cells; Club and Goblet = Club (or clara) and Goblet cells; NK = natural killer cells; NEC = neuroendocrine cells; Fibro = fibroblasts; SMCs = smooth muscle cells; aCap = alveolar capillary; gCap = general capillary; VEC = vascular endothelial cells; LEC = lymphatic endothelial cells; AT1 and AT2 = alveolar epithelial cells type 1 and 2; MegaK = megakaryocytes.

Another important role of P2X4 for lung function was discovered by Frick and colleagues. Thus, P2X4 in lamellar bodies (LBs), which are lysosome-related organelles promotes pulmonary surfactant secretion from alveolar epithelial Type II (ATII; Figure 1) cells via fusion-activated Ca^2+^ entry and subsequent fusion pore expansion [11,12,13]. Surfactant released from these LBs is necessary to increase the pulmonary compliance (i.e., the lung’s ability to stretch and expand). P2X4 is also found in secretory granules releasing mucin from secretory airway epithelial cells (goblet cells or club cells; Figure 1), where P2X4 is upregulated under conditions of chronic inflammation to augment mucin secretion [14]. Mucus clearance is an essential innate defense mechanism to keep the lung’s airways free of pathogens and particles. On the other hand, too much mucin secretion or impaired clearance of mucin can result in mucus plugging of the airways, which is in particular relevant in inflammatory lung diseases, e.g., asthma, COPD, and CF [14]. P2X4 was also demonstrated to be expressed in lysosomes and to be involved in lysosomal fusion [15]. Meanwhile, a functional role of P2X4 in endolysosomes of lung cells remains to be elucidated. Of note, high concentration of ATP in the lysosome does not activate P2X4 at low pH, only when the pH rises to 7.4, e.g., during fusion pore formation [16]. Transcriptomics data were retrieved from GEO (Accession Number GSE151674) and confirms expression of P2X4 in ATII cells and goblet cells but identify further significant expression in B cells, alveolar macrophages (AMΦ), ciliated cells, club cells, Cd209a+/Clec9a+ dendritic cells (DC), Lyve1- and Lyve1+ IMΦ (interstitial macrophages), and gCap (“general” capillary) cells which are specialized to regulate vasomotor tone and function as stem/progenitor cells in capillary homeostasis and repair (Figure 2).

## 3. TRPML3—Regulator of MMP-12 Levels in Bronchoalveolar Fluid: Early Endosomes of Alveolar Macrophages

Mucolipins are non-selective (Na^+^ > K^+^ > Ca^2+^) [17] cation channels involved in endolysosomal trafficking, fusion/fission, lysosomal pH regulation, autophagy, lysosomal exocytosis, and endocytosis. While mutations in TRPML1 cause the neurodegenerative lysosomal storage disorder mucolipidosis IV (MLIV), its relatives TRPML2 and TRPML3 are not associated with disease causing human mutations. In mice, the so called varitint-waddler mutations in TRPML3 cause deafness, circling behaviour, and coat color dilution due to hair cells in the inner ear and melanocytes in the skin dying from Ca^2+^ overload resulting from gain of function of TRPML3 mutant channels [18,19,20,21]. Hearing and vestibular functions in the *Trpml3* knockout mouse were however found to be normal [22], suggesting that loss of function of TRPML3 can be compensated or is irrelevant for normal inner ear function.

By contrast, double knockout of TRPML1 and TRPML3 in mice (*Trpml1^−/−^/Trpml3^−/−^*) causes an accelerated endolysosomal vacuolation of enterocytes and failure-to-thrive from birth to weaning [23]. This phenotype was not observed in the single knockouts [23].

Spix et al. recently reported the first bona fide single TRPML3 knockout disease phenotype [1]. In two independently generated *Trpml3* knockout mouse models, lung function measurements revealed an emphysema-like phenotype under basal conditions which strongly exacerbated under elastase treatment or tobacco smoke exposure. In both *Trpml3^−/−^* mouse models elastance (captures the elastic rigidity or the stiffness of the lungs) was reduced and compliance (captures the ease with which the lungs can be extended, i.e., lung’s ability to stretch and expand) was increased, major characteristics of emphysema, further exacerbating upon instillation of porcine pancreatic elastase or when exposed to tobacco smoke. Other lung function parameters such as tissue elasticity, inspiratory capacity, quasistatic compliance, or total lung capacity were also changed in an emphysema-like manner and the quantitative histological analysis under basal conditions and after elastase/tobacco smoke treatment revealed increased airspace enlargements, which were more pronounced in *Trpml3^−/−^* mice compared to WT mice. The authors further analysed transcriptomics data, made use of a newly generated *Trpml3^IRES-Cre/eR26-τGFP^* reporter mouse model, applied endolysosomal patch-clamp methods, and used new, isoform-selective TRPML agonists to investigate expression and function of TRPML3 in the lung where it was found to be expressed predominantly in AMΦ. Using endolysosomal patch-clamp electrophysiology, they showed functional activity in early endosomes (EE) and late endosomes/lysosomes (LE/LY) but not in recycling endosomes (RE). A cytokine/chemokine/MMP (matrix metalloproteinase) screening using Multiplex/ELISA revealed an increase in MMP-12 and MMP-8 levels in bronchoalveolar fluid (BALF) of knockout versus WT mice and in the cell culture supernatant of alveolar macrophages. In particular, high extracellular matrix MMP-12 levels are strongly associated with an emphysema/COPD phenotype. Of note, mice deficient in MMP-12 do not develop emphysema, human MMP-12 single-nucleotide polymorphisms are strongly associated with severe to very severe COPD and MMP-12 inhibitors such as AS111793 or MMP408 provide significant protection against emphysema. Importantly, the authors could demonstrate that activation of TRPML3 with a selective agonist reduced the MMP-12 levels in WT alveolar macrophage supernatants while levels were unchanged in the knockout. The authors thus provide a direct link between MMP-12 which is predominantly expressed and released by alveolar macrophages and TRPML3, the main expression of which in the lung was found to be in EE and LE/LY of alveolar macrophages (Figure 1). The authors further showed that loss of TRPML3 results in trafficking defects in the EE pathway where TRPML3 is mainly active under normal conditions (acidic pH as occurring in LE/LY blocks TRPML3 while the more neutral pH in EE increases its activity). This backlog in the EE system was postulated to result eventually in endocytosis defects and reduced extracellular clearance and reuptake of MMP-12 by alveolar macrophages. Albeit MMP-12 is also secreted predominantly from alveolar macrophages, the exact mechanisms and vesicular structures or organelles involved in its secretion remain undiscovered.

## 4. TRPV2—Critical Player in Alveolar Macrophage Phagocytosis: Cell Membrane or Early Endosome?

TRPV2 belongs to the vanilloid (V) receptor subfamily of TRP channels, is permeable for Ca^2+^ and Na^+^ (PCa/PNa = 0.9–2.9) [17], and is related to the heat, pain and capsaicin receptor TRPV1. A role for TRPV2 in COPD and phagocytosis has been proposed recently by Masubuchi and colleagues [24]. The authors found that *Trpv2* knockout mice were more susceptible to tobacco smoke exposure, showing increased airspace enlargements, which were more pronounced in *Trpv2^−/−^* mice compared to WT mice. However, no lung function data such as compliance or elastance measurements were provided by the authors to confirm the phenotype. In TRPV2 knockdown experiments using MH-S cells (murine alveolar macrophage cell line) reduced phagocytosis was claimed. Further, TRPV2 expression and phagocytosis were reduced when MH-S cells were exposed to cigarette smoke. The phagocytic function of alveolar macrophages (Figure 1) from *Trpv2^−/−^* mice was also reduced compared to WT macrophages.

Link and colleagues had shown before that zymosan-, immunoglobulin G (IgG)- and complement-mediated particle binding and phagocytosis were impaired in macrophages lacking TRPV2 [25]. Further it has been proposed that upon exposure to phagocytic substrates TRPV2 is recruited to the nascent phagosome, depolarizing the plasma membrane which then leads to increased synthesis of phosphatidylinositol-4,5-bisphosphate (PI(4,5)P_2_). PI(4,5)P_2_ then triggers actin polymerization necessary for phagocytic receptor clustering. Link et al. point however to the possibility of additional roles of TRPV2 in phagocytosis beyond substrate binding and refer to works published on the endosomal expression of TRPV2 [26,27]. Later functions of TRPV2 in phagocytosis, i.e., in (early) endosomes cannot be ruled out, e.g., a role in facilitating EE fusion and maturation.

Patch-clamp experiments performed by Saito et al. [27] suggest TRPV2 to possibly be present in EE. The pharmacological tools used by Saito et al. to characterize the currents they measured in their enlarged endosomes in HEK293 cells (stably expressing the tetracycline-inducible SDD1/VPS4B(E235Q) gene) were La^3+^ and 2-APB, which are due to their promiscuous effects of limited validity to specifically confirm TRPV2-like channel activity. State of the art endolysosomal patch-clamp experiments with more specific pharmacological tools and knockout controlled are necessary to further back up proteomics data claiming TRPV2 expression in endosomes. Transcriptomics data confirm expression of TRPV2 in AMΦ, but identify further significant expression in B cells, Cd209a+/Clec9a+ dendritic cells (DC), Lyve1- IMΦ (interstitial macrophages), NK (natural killer) cells, T cells, monocytes and neutrophils (Figure 2).

## 5. TRPM2—Protective Role in Lung Inflammation: Cell Membrane or Lysosome?

TRPM2 belongs to the melastatin (M) subfamily of TRP channels, is non-selectively permeable for Ca^2+^ and Na^+^ [17] (PCa/PNa = 0.6–0.7), and is activated by intracellular adenosine diphosphate ribose (ADPR). TRPM2 activation is further facilitated by nicotinic acid adenine dinucleotide phosphate (NAADP), cyclic ADPR, and hydrogen peroxide (H_2_O_2_) [28]. In the lung it is expressed in endothelial cells. It is also highly expressed in neutrophils, macrophages (Figure 1), mast cells, dendritic cells and other immune cells. Di and colleagues discovered a protective role of TRPM2 in lung inflammation induced by the endotoxin lipopolysaccharide (LPS). More lung infiltration by inflammatory cells, more lung edema and diminished survival for LPS-challenged *Trpm2^−/−^* compared to WT mice were observed [29]. The authors proposed that modulation of the plasma membrane potential by TRPM2 inhibits reactive oxygen species (ROS) production in phagocytes and thus prevents lung inflammatory injury induced by LPS. While TRPM2 expressed in the plasma membrane, mediating Ca^2+^ influx into cells, is undoubted, several recent studies suggest additional functional expression in lysosomes [30,31,32]. However, it remains unclear if this dual functional role of TRPM2 in both plasma membrane and lysosomes is relevant for lung disease. The same group claiming TRPM2 expression in lysosomes also explored the relevance of TRPM2 in severe asthma pathophysiology [30] since airway inflammation and asthma are linked to oxidative stress. However, Sumoza-Toledo et al. [30] found neither airway resistance nor mucus production being affected in *Trpm2^−/−^* mice. Cytokine levels, airway inflammation, allergen-induced production of IgE, or immunocyte infiltration were not affected and the authors concluded that TRPM2 might not play a significant role in allergen-mediated inflammation, at least in the model they used (ovalbumin-induced severe allergic asthma). Transcriptomics data confirm expression of TRPM2 in dendritic cells (DC), monocytes, neutrophils, and Lyve1 IMΦ with very low expression in AMΦ, (Figure 2). Compared to P2X4 and TRPV2 expression of TRPM2 is much more limited in the lung.

## 6. TRPA1—A Target for Cough, Asthma, COPD? Ca^2+^ from Lysosomes Involved?

TRPA1 is the only mammalian member of the TRPA subfamily of TRP channels is permeable for Ca^2+^ and Na^+^ (PCa/PNa = 0.84) [17], and contains 16 N-terminal ankyrin repeats in humans. TRPA1 is a chemosensor activated by a plethora of molecules including isothiocyanates, cinnamaldehyde, acrolein, nicotine, formalin, hydrogen peroxide, tear gases, and other reactive chemicals, prostaglandins, NGF, bradykinin, and histamine [6,33]. TRPA1 channel activation in the plasma membrane is well established, yet Shang et al. have recently provided pharmacological and immunocytochemical evidence that TRPA1 may also be present in endolysosomes [34]. TRPA1 was originally cloned from human fibroblasts and it is highly expressed in sensory nerve fibers. In the lung it was detected in airway epithelial and smooth muscle cells [6]. Experiments using *Trpa1^−/−^* mice and TRPA1 antagonists revealed a critical role in allergic and non-allergic airway inflammation as well as hyper-reactivity, and genetic studies in humans suggested that TRPA1 may play a role in the development of childhood asthma [35,36]. A role in cough has been proposed as well [37]. Lin and colleagues further showed that acute cigarette smoke (CS) or extract exposure rapidly activates Ca^2+^ influx in human airway smooth muscle cells (hASMC) via TRPA1, leading to myosin light-chain phosphorylation, regulating airway smooth muscle contractility. The authors speculated that this may result in further chronic pathological effects of tobacco smoke mediated by TRPA1 [38]. In their discussion the authors mention a previously published work by Rasmussen et al. [39] who found that in human airway epithelia CS triggered a rise in cytoplasmic Ca^2+^ which was inhibited by bafilomycin A1, pointing to lysosomes as potential source of the cytoplasmic Ca^2+^. At the same time, they excluded endoplasmic reticulum or mitochondria as Ca^2+^ source. Lin et al. performed their experiments with and without extracellular Ca^2+^ whereby the CS-triggered Ca^2+^ responses they observed in hASMC were absent in the latter condition, pointing to an extracellular source of the Ca^2+^. Blockers of TRPA1 or TRPA1 knockdown likewise abolished the responses. Whether the cytoplasmic Ca^2+^ rise induced by CS in airway epithelia as shown by Rasmussen et al. is TRPA1-dependent or may be mediated by other endolysosomal cation channels remains unclear. Likewise, the differences observed in hASMC and airway epithelia regarding Ca^2+^ influx across the plasma membrane versus lysosomal Ca^2+^ release upon CS exposure, remain to be further elucidated. TRPA1 was not available in the transcriptomics dataset analysed here.

## 7. BK Channel in the Lysosome—Refilling or Releasing Ca^2+^?

Large conductance voltage- and Ca^2+^-activated potassium (BK) channels, also called Maxi-K, Slo1, Kcnma1, or KCa1.1 are found on the plasma membrane, endoplasmic reticulum, Golgi, mitochondria, and lysosomes [40,41,42,43]. Indeed, in non-excitable cells BK may be mostly in intracellular organelle membranes and not in the plasma membrane [40]. In lysosomes, BK was demonstrated to be required for refilling lysosomal Ca^2+^ stores [41]. The authors showed this by using pharmacological tools and genetic ablation of BK. BK inhibition and block of refilling of the lysosomal Ca^2+^ stores resulted in cholesterol accumulation and a lysosomal storage phenotype [41]. Interestingly, Cao et al. [42] claimed rather the opposite. Thus, they suggested that Ca^2+^ release via TRPML1 activates BK activation, which then would provide a positive feedback mechanism for TRPML1 to facilitate more lysosomal Ca^2+^ release, promoting lysosomal trafficking. In agreement with Wang et al. [41] loss of BK results in lysosomal dysfunction in degradation and trafficking, and BK overexpression rescues abnormal lysosomal storage in cells from Niemann–Pick C1 patients in which TRPML1 function is impaired [42]. Furthermore, the same group showed that in macrophages lysosomal BK channels regulate large particle phagocytosis in cooperation with TRPML1, through modulating lysosomal exocytosis [44]. In the lung, BK channels are highly expressed in airway smooth muscle cells (SMCs) [45] and Goldklang et al. suggested that activation of BK in hASCM may have a benefit in treating asthma by reducing inflammatory cell infiltration and airway resistance.

BK is also found in lung fibroblasts [46]. Fibroblasts from patients with idiopathic pulmonary fibrosis (IPF) exhibit increased expression of KCNMB1, which codes for a β-subunit of BK. Scruggs et al. [46] further demonstrated that this upregulation of KCNMB1 contributes to increased BK channel activity in IPF fibroblasts, fostering differentiation of fibroblasts into myofibroblasts, a critical step in the development of fibrotic disorders such as IPF. In this work, BK activity was demonstrated in the plasma membrane of fibroblasts using the whole-cell patch-clamp technique, but BK activation was followed by a rise in intracellular Ca^2+^, promoting differentiation into myofibroblasts. While the authors only speculate about the source of the intracellular Ca^2+^, they do not exclude intracellular Ca^2+^ stores playing a role. In sum, while evidence is accumulating for important functional roles of BK in lysosomes, a connection between lysosomal BK and lung disease is missing so far. Transcriptomics data confirm expression of BK in SMCs and in fibroblasts, in particular in Lgr5+Lgr6+ fibroblasts (Figure 2) and reveal very little expression elsewhere in the lung.

## 8. Discussion and Conclusions

Clearly, more needs to be performed to establish and further validate TRPV2, TRPM2, and TRPA1 as ion channels in endolysosomes. None of the currently published studies made use of the now well established endolysosomal patch-clamp technique [47] to directly demonstrate functional activity in endosomal or lysosomal membranes. While involvement of these channels in certain aspects of lung function and pathophysiology is well documented, clear links to endolysosomal channel activity are not confirmed. Only P2X4 and its role in surfactant release from lamellar bodies, lysosome-related organelles is well proven; likewise, the recent study of TRPML3 and its role for emphysema formation is strongly linked to its expression in endolysosomes. Plasma membrane expression of TRPML3 in endogenous cell systems including alveolar macrophages could not be confirmed [1]. By contrast, while the patch-clamp evidence for BK in endolysosomes is very convincing, potential roles in lung function seem not connected to endolysosomes.

Other cation channels which are strongly linked to endolysosomes are TRPML2 (Rab4+ and Rab11+ RE, EE and LE/LY), TRPML1 (LE/LY), TPC1 (EE/LE), and TPC2 (LE/LY). These channels have not been explored in the context of lung disease yet but may be interesting candidates for relevant roles in lung physiology and pathophysiology such as TRPML3. Their roles in autophagy [48,49,50,51,52], release of inflammatory mediators [53,54,55], endolysosomal trafficking, exocytosis and phagocytosis [56,57,58,59,60,61], and their presence in the lung highly suggest functional relevant roles in lung diseases.

Multiple links exist between autophagy and lung disease. Activated autophagy reportedly protects the lung from damage by decreasing cellular senescence, inhibiting myofibroblast differentiation, and reducing infection. On the other hand, increased autophagy contributes to increased COPD pathogenesis by promoting epithelial cell death, and expression of LC3B-II, Atg4B, Atg5, Atg12, and Atg7 was significantly increased in COPD lungs. Furthermore, Monick et al. reported defective autophagy in alveolar macrophages of smokers, suggesting that impaired delivery of bacteria to lysosomes may lead to recurrent infections in patients with COPD [62,63,64,65,66]. In addition to autophagy, also lysosomal dysfunction such as in lysosomal storage disorders can be associated with lung disease: e.g., interstitial lung disease (ILD) in Gaucher’s disease (GD) and in Niemann–Pick disease Types A, B, and C, obstructive airway disease in Fabry disease (FD), chronic respiratory failure secondary to muscle weakness in Pompe disease, and both obstructive and ventilator impairment in mucopolysaccharidosis (MPS) and mucolipidosis (ML) [67,68]. Pulmonary involvement occurs in all three types of Niemann–Pick disease but most frequently in Type B [67] with 25% of the deaths caused by respiratory disease. Most of these patients die from ILD, which may or may not be associated with infectious pneumonia or, more rarely, pulmonary embolism [67]. Pulmonary involvement in NPC1 may also be more common than previously reported [69]. In Fabry disease, respiratory symptoms are mostly COPD-like, ILD is rare. In GD, the most common pulmonary function abnormalities are: reduced functional residual capacity, decreased carbon monoxide transfer coefficient and reduced total lung capacity [67]. In a cohort of 95 patients with GD 68% had some pulmonary dysfunction. Pulmonary dysfunction is reportedly also a frequent problem in most forms of MPS and ML [68]. These examples highlight the relevance of a healthy endolysosomal system for lung function and encourage further investigation of central players in the endolysosomal system such as TRPML1 or TPC2 in the context of lung physiology and disease.

Furthermore, infectious lung diseases are an increasing health issue and diseases such as tuberculosis, influenza, MERS-CoV or SARS-CoV demonstrate the importance but also the vulnerability of the endolysosomal system. While the role of endolysosomal cation channels in infectious diseases is reviewed elsewhere [70], it is tempting to speculate that TPC or TRPML channel dysfunction or hyperactivity may impact lung function and lung disease e.g., COPD or pulmonary fibrosis progression, during infections with respiratory pathogens, given their roles in phagocytosis, exocytosis and intracellular trafficking. Indeed, there is evidence that pharmacological blockage or genetic ablation of TPCs or TRPMLs interferes with the infectivity of a range of bacteria, bacterial toxins, and viruses including Ebola and SARS-CoV2, i.e., reducing their infectivity and disease severity [71,72,73,74]. However, currently there are no experiments available confirming that blockage or interference with, e.g., TRPML2 or TPC1/2 reduces influenza A virus or SARS-CoV2 infectivity in vivo. Neither seem the currently available data sufficiently knockout controlled. Whether patients with COPD, pulmonary fibrosis, or other chronic lung diseases may benefit from TRPML/TPC block during infections remains likewise unexplored. Vice versa, as in the case of TRPML3, loss of the channel rather exacerbates lung disease for other reasons such as defects in inflammatory mediator endocytosis. At least in the case of TRPML3, the recently published data on emphysema due to channel loss suggest that block or loss of TRPML3 is sufficient to induce emphysema formation or exacerbation due to defective MMP-12 endocytosis and neutralization. Such a negative impact on lung function and disease remains to be explored for the related TRPML channels and TPCs.
